# Consistent Pattern of Local Adaptation during an Experimental Heat Wave in a Pipefish-Trematode Host-Parasite System

**DOI:** 10.1371/journal.pone.0030658

**Published:** 2012-01-27

**Authors:** Susanne H. Landis, Martin Kalbe, Thorsten B. H. Reusch, Olivia Roth

**Affiliations:** 1 Leibniz Institute of Marine Sciences (IFM-GEOMAR), Evolutionary Ecology of Marine Fishes, Kiel, Germany; 2 Max-Planck-Institute for Evolutionary Biology, Dept. Evolutionary Ecology, Plön, Germany; University of Minnesota, United States of America

## Abstract

Extreme climate events such as heat waves are expected to increase in frequency under global change. As one indirect effect, they can alter magnitude and direction of species interactions, for example those between hosts and parasites. We simulated a summer heat wave to investigate how a changing environment affects the interaction between the broad-nosed pipefish (*Syngnathus typhle*) as a host and its digenean trematode parasite (*Cryptocotyle lingua*). In a fully reciprocal laboratory infection experiment, pipefish from three different coastal locations were exposed to sympatric and allopatric trematode cercariae. In order to examine whether an extreme climatic event disrupts patterns of locally adapted host-parasite combinations we measured the parasite's transmission success as well as the host's adaptive and innate immune defence under control and heat wave conditions. Independent of temperature, sympatric cercariae were always more successful than allopatric ones, indicating that parasites are locally adapted to their hosts. Hosts suffered from heat stress as suggested by fewer cells of the adaptive immune system (lymphocytes) compared to the same groups that were kept at 18°C. However, the proportion of the innate immune cells (monocytes) was higher in the 18°C water. Contrary to our expectations, no interaction between host immune defence, parasite infectivity and temperature stress were found, nor did the pattern of local adaptation change due to increased water temperature. Thus, in this host-parasite interaction, the sympatric parasite keeps ahead of the coevolutionary dynamics across sites, even under increasing temperatures as expected under marine global warming.

## Introduction

The interaction between hosts and parasites is often conceptualized as an arms race where hosts and parasites need to adapt and counter adapt permanently to keep up with the evolution of the respective antagonist [1], [2]. Parasite populations are expected to adapt to the locally most common host genotypes [3], [4] while at an individual level matching host×parasite genotypic interactions provide the genetic basis for this adaptation [5]–[9]. Given the large population sizes and/or short generation times, many parasite species have a greater evolutionary potential than their hosts. Consequently, hosts are expected to lag behind in this arms race, resulting in parasite populations being more often adapted to their sympatric hosts than vice versa [10]. However, as the infection outcome depends on the combinatorial effect of specific genotypes on both sides [11], even certain allopatric hosts can be less resistant than certain sympatric ones [4]. In addition, the local environment often determines the outcome of coevolutionary dynamics. Hence, any process of local adaptation of host or parasite is confined to a locality and therefore may differ between regions at the same time point [12]–[14]. If the environment changes, for example by imposing warmer temperature conditions, any such local adaptation process can be disrupted, whereas this disruption can again be spatially restricted.

In this context, it is unfortunate that most empirical evidence for local adaptation and genotype×genotype interactions stem from controlled laboratory experiments representing stable environmental conditions [15], [16], while studies about host-parasite interactions that incorporate environmental change by global warming are rare (but see [17]–[19]). Extreme climate events such as heat waves and storms affect ecosystems, populations and species interactions [20]–[22], and are expected to rise in frequency [15], [23], [24]. Whereas global sea surface temperatures have increased by an average of 0.6°C from 1980–1999 [20], regional increases can be much higher, in particular in our study area, the Baltic and North Sea [25].

As first order effects, warming and other globally related changes are expected to accelerate pathogen developmental rates, transmission and number of generations per year [26]. These factors may then contribute to the emergence of diseases in the marine environment. Moreover, since a host's overall condition determines its ability to sustain an immune response [27], and as many alternations associated with global change impose additional stress on the organism [28], [29], immunocompetence may generally decrease under heat stress [24].

Here, we investigated experimentally the effect of an extreme climatic event, that simulated the 2003 European heat wave [21], on host-parasite combinations of the broad-nosed pipefish *Syngnathus typhle* and its digenean trematode *Cryptocotyle lingua*. First order effects on parasite infectivity and host immunity under global warming conditions can be expected as sea surface temperature is known to have effects on *Syngathus* species, for example higher reproduction rates and higher abundances have been shown [30], [31]. We hypothesized that the digenean trematode parasite *C. lingua* with its complex, three-host life cycle, may benefit from warm water because the immune defence of the invertebrate intermediate host may decrease under these conditions [17]. In contrast, the proliferation of trematode infective stages (cercariae) as well as their activity has been suggested to be increased until a temperature threshold [32], [33]. To test if the parasite will benefit from the warming, fully reciprocal cross infections of hosts and parasites from three locally distinct populations were performed to assess whether or not consistent patterns of local adaptation exist. Due to the greater evolutionary potential of parasites we expected to find local parasite adaptation in all tested populations. To do so, we compared sympatric with allopatric host-parasite combinations by applying a specificity index [34]. Furthermore, we examined if environmental change can disrupt locally adapted host-parasite combinations and whether the pattern of local adaptation is stable enough to endure the impact of a simulated heat wave. This was tested by examining the three-way interaction between host, parasite and temperature.

## Materials and Methods

### Model organisms

The broad-nosed pipefish *Syngnathus typhle* is sex-role reversed and lives in shallow eelgrass (*Zostera marina*) meadows along the coasts of Europe and has a lifespan of two or more years [30], [35]. *S. typhle* is regularly infected by a variety of parasite species [36], including our study species, the digenean trematode *Cryptocotyle lingua*. This generalist parasite shows a broad diversity of possible second intermediate hosts, but most of them are small coastal fishes [37]. Throughout European coastal waters *C. lingua* shows a high diversity where the majority of its European haplotypes can only be observed once [38]. Its larval cercarial stages penetrate the skin and lead to visible black spots (metacercariae). In the field we observed varying intensities (between 0 and 30 metacercariae) of infection with *C. lingua*. The complex life cycle of *C. lingua* includes three hosts: snails and fishes as first and second intermediate hosts respectively, and gulls as final hosts. The sexual reproduction takes place in the gull and parasite eggs are then shed in the water, with the gull's faeces. These eggs are then eaten by the first intermediate host, generally by the snail *Littorina littorea*. In the snail the eggs hatch into miracidia and penetrate the gut wall where they metamorphose into sporocysts [39]. In the sporocyst the rediae are formed and from its germ cells cercariae will be produced [40]. The cercariae are shed into the water, where the free-swimming forms search and penetrate the second intermediate host, generally fish, in which they transform into encysted metacercariae [40]. This complex life cycle excludes direct transmission from one fish host to another [40] and makes it a suitable model for laboratory infection.

### Animal collection and preparation

We collected snails (*Littorina littorea*) and uninfected (no visible black spots) pipefish (*Syngnathus typhle*) in July and mid August 2010 at three different locations in North Europe in shallow water with eelgrass meadows (5^th^ and 6^th^ of July at Geltinger Bucht, Germany, N 54°75,57′; E 9°87,66′; on the 13^th^–15^h^ of August in Fiskebäckskil, Sweden, N 58°24.80′; E 11°44.62′; at the 16^th^ of August at Doverodde, Denmark, N 56°71.90′ E 8°46.38′) by snorkelling using hand nets. Water temperature at the locations was between 14° and 18°C. We collected around 500 snails at each location at the shore on rocks, preferably at places with gull faeces to raise the possibility of an infection with *C. lingua*. From each location we collected between 90 and 100 juvenile pipefish, sex was mostly not identifiable yet. Before the experiment we let the fish acclimatise in 200 l barrels filled with 18°C water and artificial eelgrass for at least 15 days, and fed them ad libitum with live *Mysis* and frozen *Artemia*. Snails from the same location were kept in 20 l aquaria with constant aeration and fed ad libitium with *Ulva* sp. algae. Both, fish and snails were kept at 18°C and summer light conditions (day: 15 h, night: 9h).

### Experimental design

We aimed to gain information about how a simulated heat wave, as environmental effect (E), affects a host-parasite interaction using a full factorial infection matrix of sympatric and allopatric host (H) and parasite (P) combinations (i.e. H×P×E). The pipefish were either kept at 18°C normal condition, or experienced a heat wave by increasing the water temperature to 25°C (incremental temperature increase of 1°C per day for 7 days), as it occurred in the Baltic Sea in 2003 [23], [41]. After reaching 25°C, this temperature was kept constant the following three weeks until the end of the experiment at either 25°C (high temperature) or 18°C (low temperature) to follow the 2003 summer heat wave situation in the Baltic Sea [21].

Pipefish from one population and the same temperature group were kept in a 200 l tank filled with aerated natural seawater (25 PSU) and artificial eelgrass before the start of the experiment. Original salinities were 16 PSU for German fish, 24 PSU for Danish fish and 27 PSU for Swedish fish, during the experiment salinity was kept rather high to prevent infections with *Vibrio* [42]. The fish were fed ad libitum with *Mysis* and *Artemia*. All snails were constantly kept at 18°C. In order to trigger cercariae release, the snails were removed from the aquaria and placed individually in 50 ml Falcon tubes under a lamp. The released cercariae were checked under the stereomicroscope to confirm the species identity and the concentration was assessed. Cercariae from the same snail population were pooled and the concentration adjusted for the three different populations. This cercariae solution was then used for infection.

The experiment started at the 1^st^ of September when all fish were tagged with Visible Implant Elastomer (from NMT) with an individual colour code. For infection, pipefish from both temperature treatments (18°C and 25°C) were placed in 5 l tanks with the according acclimatisation temperature, in pairs of two as keeping fish singly imposes too much stress on pipefish. The fully reciprocal design included 3 hosts (DK, GER, SWE)×4 parasites (DK, GER, SWE+control)×2 temperatures (18°C and 25°C) = 24 treatments in total. Per treatment we used 10 replicates, which translated to a total of 240 experimental fish (24 treatments×10 replicates). During the next five days all fish were exposed daily to seawater containing cercariae or to seawater without cercariae as control treatment. Summed over five days the total dose were 80 *C. lingua* cercariae, coming from between 24 and 30 snails/population (with the following daily concentrations for all three locations [cercariae/aquaria]: 14, 26, 14, 13, 13).

During the infection week, 22 of 40 fish (infected and control) from the Swedish population in the normal water group (18°C) died, suffering from a fungal disease. 11 of the remaining fish died the following week. We therefore had to exclude this treatment group (Swedish fish, 18°C) from the data analysis.

After infection, 5–6 pipefish individuals (variation due to the Swedish fish) from the same origin and from the same temperature group were randomly placed together into larger, 80 l tanks (N = 36) in a circulating water system at either 18°C (control) or 25°C (heat wave). This minimized a possible stress response of the fish, confounding the latter immune measurements. Aquaria were arranged randomly and the two temperature groups (18°C and 25°C) were supplied with the same water (again at 25 PSU). The fish were fed twice a day with live *Mysis* and frozen *Artemia* and had artificial eelgrass and aeration in their tanks. After seven days the immune response of the fish and the transmission success of the parasite were measured.

To measure immune response the fish were killed and dissected in a random order during three subsequent days. We focused on the head kidney which is a major fish immune organ [43], [44], in particular for Syngnathids, as they lack a spleen [45]. We measured the proportion of lymphocytes and monocytes by means of flow cytometry, using a FACSCalibur and CellQuest Pro Software (both BD) to gain information about the adaptive and innate immune system. As a parameter for the activity of the adaptive immune system, the relative number of lymphocytes in the G_2-M_ phase and S phase of the cell proliferation cycle were determined in contrast to the resting phase G_01_ [43].

Before the experiment we dissected over 50 naturally infected fish from the field to find the ratio of metacercariae in skin and organs or muscle tissue. In all cases we only could find metacercariae (also visible as “black spot”) in the skin. The appearance of a “black spot” therefore is to our understanding a suitable approximation for the success of the parasite entering the host. To assess the parasite's infectivity we counted metacercarial spots [46].

All experiments were performed according to current national legislation on Animal Welfare. Prof. Dr. Gerhard Schultheiss from the Ministerium für Landwirtschaft, Umwelt und ländliche Räume des Landes Schleswig-Holstein approved to perform the experiments with the permit number 187, named “Effects of global change on immunological interaction of pipefish and their natural parasites” described in this manuscript.

### Data analysis

#### a) Specificity index to detect local adaptation

We tested for local adaptation by calculating a specificity index after [34]. The specificity index is derived by subtracting the mean transmission rate (number of metacercariae) of the allopatric from the sympatric measurements. It thus incorporates both, host resistance and parasite infection success by allowing comparisons between host populations and parasite populations respectively: The higher the metacercariae number, the higher the infection success. If the specificity index is positive, the sympatric parasites have a higher infectivity than the allopatric ones. If the value is smaller than zero, the allopatric parasites are more infective, showing a higher host resistance to the sympatric parasite. However, if specificity indices are not differentiable from zero, no local adaptation can be detected.

The data was Box Cox transformed ((((x+2)^2^)−1)/(−0.07)) to reach normal distribution. If the index values differed from zero (tested with a one sample t-test, corrected for false discovery rate (FDR)), sympatric values varied from allopatric ones and either host or parasite were locally adapted.

#### b) Host×parasite×environment

To characterize the immune response of pipefish hosts, two sorts of immune measurements were performed: 1.) *Cell count*: the proportion of blood cells (lymphocytes or monocytes relative to all live cells) that were extracted from the head kidney and 2.) *Cell cycle stage*: the abundance of particular cell cycle stages reflecting the activity of the lymphocytes (G_01_, S and G_2-M_ stage) were determined. We combined the proportions of S and G_2-M_ cells in both stages together as both reflect activity in contrast to cells in the G_01_ stage. Before the analysis the cell count data was Box Cox transformed (prop. lymphocytes: ((x^0.4^−1)/−1.345)), prop. monocytes: ((x^2^−1)/1.003))) to reach normal distribution. Infectivity of the parasite was measured in counted metacercariae in the skin after infection.

We then performed two-way ANOVAs for five response variables (number of metacercariae, proportion of lymphocytes, proportion of monocytes, proportion of cells in G_01_ stage, proportion of cells in S - G_2-M_ stage) with temperature (18°C vs. 25°C) and host-parasite combination (sympatric, allopatric, control) as fixed factors. We corrected the ANOVAs for multiple testing with Bonferroni corrections. Significant effects were further analyzed with a Tukey HSD test based on the ANOVAs. The tank effect was included in the analysis as random factor in a Restricted Maximum Likelihood model (REML).

As infection took place keeping two fish in the same aquarium, we verified whether using both fish as independent replicates affected the outcome of the analysis. For this we performed the same ANOVAs as described above and reduced the replicate size to one fish per aquarium by randomly choosing one of the two fish. In a second analysis we used both fish as independent replicates and ran the same analysis again. All effects were consistent in both statistical analyses, independent of whether one or both fish per aquarium were analyzed, or whether all fish were analysed as single replicates.

## Results

### Local adaptation

For all three host-parasite populations tested we found positive values for the specificity index, indicating local parasite adaptation. These effects were independent of temperature. Not all specificity indices were significantly different from zero (Table 1), but for each host-parasite combination, at least one specificity index differentiated significantly from zero. This indicates a clear pattern of local adaptation of the parasite to its local host. This pattern persists even if we allocate the host-parasite interaction into a new, warmer environment by imposing a heat wave.

**Table 1:** Specificity index calculated by subtracting the allopatric mean of the metacerariae number from the sympatric one per population (Denmark, Germany, Sweden) and temperature treatment (25°C, 18°C) to assess local adaptation from either the host's side (resistance) or the parasite's side (infectivity).

25°C	Si Index	t/V	df	p	FDR 0.05	Significance
*Denmark*						
resistance	2.8	9.37	8	0.0001	0.01667	sym>allo
infectivity	2.1	0.206	8	0.84	0.05	n.s
*Germany*						
resistance	4.8	6.94	7	0.0002	0.033	sym>allo
infectivity	4.5	2.64	7	0.031	0.033	sym>allo
*Sweden*						
resistance	3.6	0.303	6	0.77	0.05	n.s
infectivity	4.6	13.6	6	0.00001	0.01667	sym>allo

18°C		t/V	df	p	FDR 0.05	Significance
*Denmark*						
resistance	2	8.8	2	0.012	0.025	sym>allo
infectivity	0.8	−0.42	2	0.72	0.05	n.s
*Germany*						
resistance	−0.7	−1.2	8	0.26	0.05	n.s
infectivity	0.2	9.4	8	0.0001	0.025	sym>allo

If the value is higher than zero, parasite local adaptation can be detected. The SI index was analyzed with a one sample t-test, corrected for false discovery rate (FDR).

### Host × parasite × environment interaction

**Figure 1:**
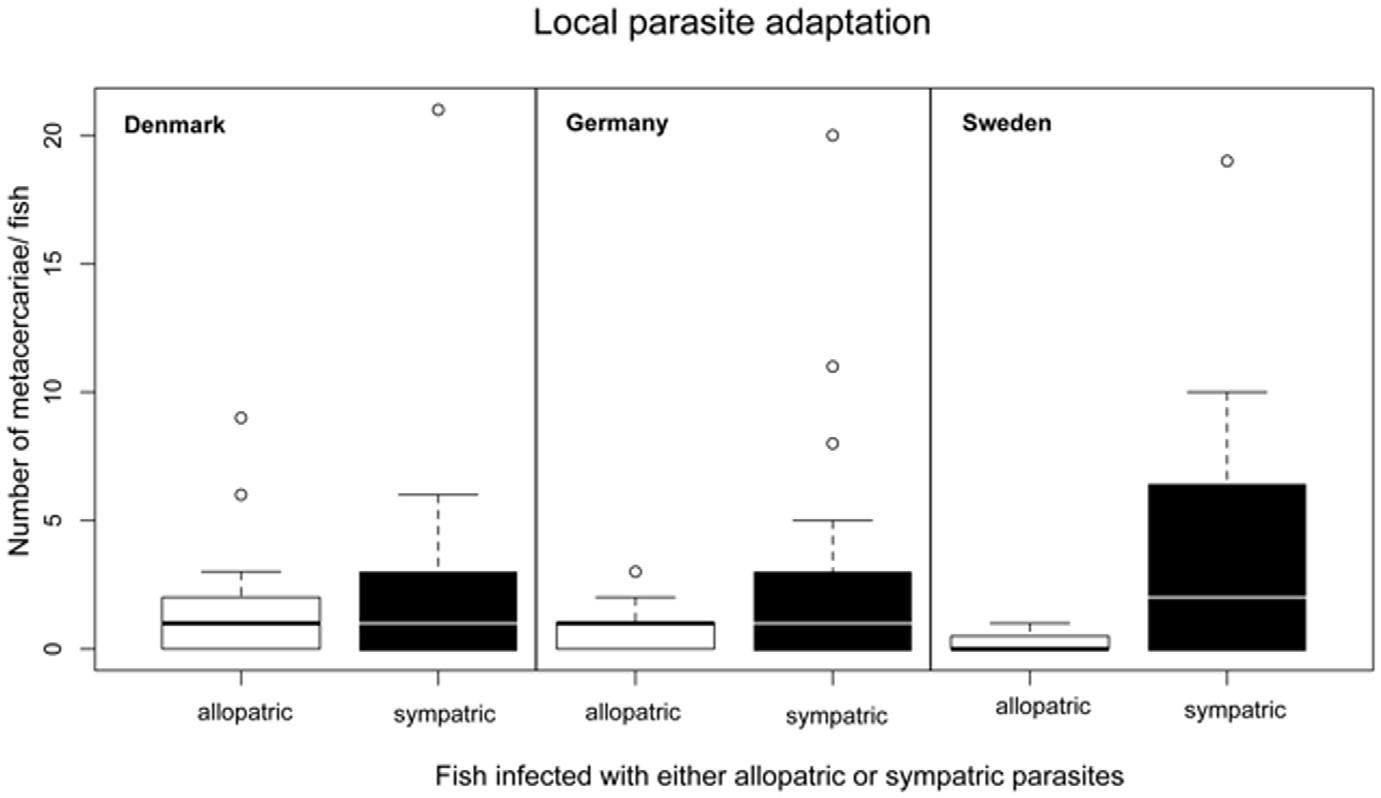
Local parasite adaptation: Counted metacercariae per fish per population, either infected with sympatric (black boxes) or allopatric (white boxes) parasites. Boxplot shows median, upper and lower quartile, minimum and maximum.

**Figure 2:**
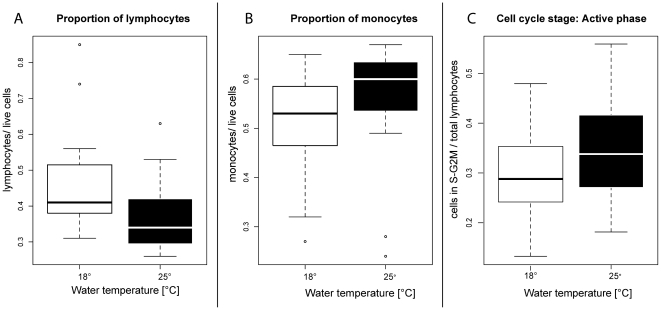
Effect of temperature on proportion of lymphocytes (A), proportion of monocytes (B) and activity of lymphocytes (C) for head kidney cells of *S. typhle* experienced a heat wave (black boxes, 25°C) and control treatment (white boxes, 18°C). Boxplot shows median, upper and lower quartile, minimum and maximum.

The outcome of the infection (number of metacercariae) is higher, when sympatric hosts and parasites meet (ANOVA with subsequent Tukey HSD, p adj. = 0.003, Figure 1, Table 2). On the other hand we found that the heat wave altered important elements of the pipefish immune defence compared to the control condition of 18°C (Table 2 for details). The relative number of lymphocytes decreased (ANOVA with subsequent Tukey HSD, p adj. = 0.0001) in warm water indicating a downregulation of the adaptive immunity pathway. In contrast, the number of monocytes that are characteristic for innate immunity increased (ANOVA with subsequent Tukey HSD, p adj.<0.0001) in warm water compared to the control group. The water temperature also had a significant effect on the proliferation of lymphocytes. We found a significantly higher proportion of cells in S-G_2-M_ stage in warm water (ANOVA with subsequent Tukey HSD, p adj. = 0.007) than in the control group and consequently more cells in resting stage G_01_ in the control treatment of 18°C than in the warm water (ANOVA with subsequent Tukey HSD p adj. = 0.02, Figure 2).

**Table 2:** ANOVAs with Bonferroni correction (α = 0.01) with response variables: number of metacercariae, proportion of lymphocytes and monocytes, proportion of cells in G_01_, S-G_2-M_ stage, with fixed factors: temperature treatment (25°C, 18°C) and host – parasite combination (allopatric, sympatric, control).

ANOVAsResponse variables	Fixed factors	Df	F value	Pr(>F)	Bonferroni significant
**Metacercariae**	Temperature	1	2.82	0.095	
	H – P combination	2	9.29	0.0002	*
	Temp*Combination	2	3	0.053	
	Residuals	122			
**Proportion of lymphocytes**	Temperature	1	29.089	3.44 E-07	*
	H – P combination	2	2.7474	0.068	
	Temp*Combination	2	0.0287	0.982	
	Residuals	122			
**Proportion of monocytes**	Temperature	1	13.476	0.0004	*
	H – P combination	2	3.1371	0.047	
	Temp*Combination	2	0.018	0.981	
	Residuals	122			
**Proportion of G_01_**	Temperature	1	7.0737	0.008	*
	H – P combination	2	1.4573	0.237	
	Temp*Combination	2	0.6674	0.515	
	Residuals	122			
**Proportion of S-G_2M_ stage**	Temperature	1	7.4235	0.007	*
	H – P combination	2	2.0822	0.129	
	Temp*Combination	2	1.3394	0.265	
	Residuals	122			

## Discussion

In this study we examined whether increasing temperature conditions, simulating an extreme event of climate change such as the 2003 European heat wave, alter sympatric and allopatric host-parasite interactions. To this end, we measured the infectivity of the parasite (i.e. the number of metacercariae in each pipefish host) and the immune defence of the host (i.e. the relative number of monocytes and lymphocytes in the head kidney and the cell cycle stage of the lymphocytes). As one prerequisite for our major study objective, we found that sympatric parasite isolates always were most successful in infecting local compared to allopatric hosts. This result suggests that the parasites were locally adapted to their hosts (Figure 1). Potential pre-exposure of the fish to their local parasite in the field would be expected to result in a stronger immune response against the sympatric parasite and hence, in a potentially lower infectivity. Our results thus cannot be explained by immune priming, but rather are an argument for a strong pattern of local adaptation.

### Temperature effects on host

Pipefish from all three populations and parasite treatments (sympatric, allopatric and control), when exposed to a simulated heat wave had fewer cells of the adaptive immune system (lymphocytes) compared to the same groups that were kept at 18°C. The proportion of the innate immune cells (monocytes) was higher in all 3 treatment groups in the 18°C water compared to individuals kept in 25°C water (Figure 2). Moreover, cells of the adaptive immune system proliferated faster in the warm water. Compared to the constitutive and fast activation of the innate immune response the activation of the adaptive immune system is both slow and costly [47]–[49]. Innate and adaptive immune pathways depend in several steps on each other which may result in resource allocation trade-offs in either the fast innate or the costly specific immune response [50]. Our findings of a higher activity of lymphocytes (Figure 2) under a heat wave compared to normal temperature combined with a lower proportion of lymphocytes suggest that the adaptive immune response is delayed under increasing environmental temperatures. Alternatively, a higher bacteria prevalence and virulence in warm waters could explain the decreased lymphocyte count at an increased proliferation, as cells may leave the head kidney to fight bacteria infections in the periphery [51].

### Temperature effects on parasite

Contrary to other studies, in which infectivity positively correlated with increasing temperature [52], [53], the temperature treatment had no effect on the infectivity of *C. lingua*. Infection patterns were consistent across treatments and not influenced by the heat wave, which indicates that local adaptation between genotypes was more important than first order temperature effects on the parasite. Due to the complex life cycle, the infectivity of *C. lingua* depends upon various biotic and abiotic factors acting on all stages. For example first intermediate hosts (invertebrates) of *C. lingua* were shown to have a decreased immune response under a scenario of global change [17]. The cercariae production is likely to be higher in warmer waters [32], However, a trade-off between cercarial activity and longevity was identified recently in 20°–25°C warm water in a different species [33].

### Local adaptation

*C. lingua* is locally adapted to its host as more parasites from the same location (sympatric) infected hosts than from a different (allopatric) location. This is of particular importance as *C. lingua* is a generalist parasite [36], with a broad range of different host species that has a mobile final bird host. Local adaptation is counterintuitive in generalists as they need to keep the potential to infect several hosts [54]. However, due to its final bird host and thus high geographic dispersal, it seems that *C. lingua* might be ahead in the evolutionary arms race, possibly due to a higher gene flow between adjacent parasite populations and therefore the parasite may profit from an inflow of new alleles that promote adaptation to new host resistance alleles [55].

Our heat wave experimentally displaced the different host – parasite combinations to a new thermal environment. If patterns of local adaptation were strongly influenced by the environment, as proposed by the geographic mosaic theory [12], [13], we expect that at least some patterns would alter in magnitude or sign. Yet, regardless of the temperature conditions all combinations of allopatric and sympatric hosts and parasites revealed a consistent sign of local adaptation, with sympatric parasites infecting their hosts better than allopatric ones.

### Conclusions

This is, to the best of our knowledge, the first study that investigates immune parameters of the adaptive/innate response in a teleost under a realistic heat stress scenario to assess possible immune system suppression under global change. Our latter findings of a marked alteration under a realistic scenario of temperature stress highlight the importance of studies on ecological immunity [56]–[58] to be included as response variables in global change research. We are also not aware of any study that measures the outcome of combinations of coevolved populations of hosts and parasites under an extreme event associated with global change. Because we only tested two environmental conditions, over a relatively short time, we cannot rule out that the patterns of local adaptation observed here are changed by the multitude of factors that are affected by global change.
